# Periconceptional Maternal Mediterranean Diet Is Associated With Favorable Offspring Behaviors and Altered CpG Methylation of Imprinted Genes

**DOI:** 10.3389/fcell.2018.00107

**Published:** 2018-09-07

**Authors:** John S. House, Michelle Mendez, Rachel L. Maguire, Sarah Gonzalez-Nahm, Zhiqing Huang, Julie Daniels, Susan K. Murphy, Bernard F. Fuemmeler, Fred A. Wright, Cathrine Hoyo

**Affiliations:** ^1^Bioinformatics Research Center, North Carolina State University, Raleigh, NC, United States; ^2^Center for Human Health and the Environment, North Carolina State University, Raleigh, NC, United States; ^3^Department of Environmental and Occupational Health, University of Pittsburgh, Pittsburgh, PA, United States; ^4^Department of Biological Sciences, North Carolina State University, Raleigh, NC, United States; ^5^Department of Health, Behavior and Society, Johns Hopkins Bloomberg School of Public Health, Baltimore, MD, United States; ^6^Department of Obstetrics and Gynecology, Duke University Medical Center, Durham, NC, United States; ^7^Department of Epidemiology, University of North Carolina at Chapel Hill, Chapel Hill, NC, United States; ^8^Department of Health Behavior and Policy, Virginia Commonwealth University, Richmond, VA, United States; ^9^Department of Statistics, North Carolina State University, Raleigh, NC, United States

**Keywords:** maternal diet, neuro-development, cord-blood methylation, child behavior disorders, ADHD-attention deficit disorder, autism spectrum disorder, epigenetics, imprinted genes

## Abstract

**Background:** Maternal diet during pregnancy has been shown to influence the child neuro-developmental outcomes. Studies examining effects of dietary patterns on offspring behavior are sparse.

**Objective:** Determine if maternal adherence to a Mediterranean diet is associated with child behavioral outcomes assessed early in life, and to evaluate the role of differentially methylated regions (DMRs) regulating genomically imprinted genes in these associations.

**Methods:** Among 325 mother/infant pairs, we used regression models to evaluate the association between tertiles of maternal periconceptional Mediterranean diet adherence (MDA) scores derived from a Food Frequency Questionnaire, and social and emotional scores derived from the Infant Toddler Social and Emotional Assessment (ITSEA) questionnaire in the second year of life. Methylation of nine genomically imprinted genes was measured to determine if MDA was associated with CpG methylation.

**Results:** Child depression was inversely associated with maternal MDA (Bonferroni-corrected *p* = 0.041). While controlling for false-discovery, compared to offspring of women with the lowest MDA tertile, those with MDA scores in middle and high MDA tertiles had decreased odds for *atypical* behaviors [OR (95% CI) = 0.40 (0.20, 0.78) for middle and 0.40 (0.17, 0.92) for highest tertile], for *maladaptive* behaviors [0.37 (0.18, 0.72) for middle tertile and 0.42 (0.18, 0.95) for highest tertile] and for an index of *autism spectrum disorder* behaviors [0.46 (0.23, 0.90) for middle and 0.35 (0.15, 0.80) for highest tertile]. Offspring of women with the highest MDA tertile were less likely to exhibit *depressive* [OR = 0.28 (0.12, 0.64)] and *anxiety* [0.42 (0.18, 0.97)] behaviors and increased odds of *social relatedness* [2.31 (1.04, 5.19)] behaviors when compared to low MDA mothers. Some associations varied by sex. Perinatal MDA score was associated with methylation differences for imprinted control regions of *PEG10/SGCE* [females: Beta (95% CI) = 1.66 (0.52, 2.80) – Bonferroni-corrected *p* = 0.048; males: -0.56 (-1.13, -0.00)], as well as both *MEG3* and *IGF2* in males [0.97 (0.00, 1.94)] and -0.92 (-1.65, -0.19) respectively.

**Conclusion:** In this ethnically diverse cohort, maternal adherence to a Mediterranean diet in early pregnancy was associated with favorable neurobehavioral outcomes in early childhood and with sex-dependent methylation differences of *MEG3*, *IGF2*, and *SGCE/PEG10* DMRs.

## Introduction

Numerous prenatal environmental exposures including smoking, stress, maternal obesity, and dietary factors have been associated with health and neurodevelopmental outcomes in children (Huizink et al., 2002; Wiebe et al., 2009; Steenweg-de Graaff et al., 2012; Gartstein and Skinner, 2017), with some exposures conferring life-long associations (House et al., 2016; Wyss et al., 2017). In a recent meta-analysis, pre-pregnancy obesity was estimated to be associated with a 51% increased risk of any neurodevelopmental impairment and a 62% increased risk of attention deficit hyperactivity disorder (ADHD) symptoms or ADHD related impairments (Sanchez et al., 2017). Prenatal nutrition may also have lasting effects on child neurodevelopment (Anjos et al., 2013; Lyall et al., 2014). While extensive research concerning maternal obesity, gestational weight gain, and child behavior has been conducted [for reviews see (Rivera et al., 2015; Contu and Hawkes, 2017; Sanchez et al., 2017)], less is known about particular types of maternal diets in humans and their effects on early childhood behavior.

Numerous studies suggest that there may be beneficial effects of maternal diet in pregnancy on child neurodevelopment, albeit, again with mixed results (Borge et al., 2017). Intakes of antioxidants, n-3 fatty acids, and foods rich in these nutrients have been associated with child developmental outcomes with inconsistent findings (Guxens et al., 2012; Brew et al., 2015; Delgado-Noguera et al., 2015; Emmett et al., 2015; Bolduc et al., 2016; Catena et al., 2016; Ishikawa et al., 2016; Julvez et al., 2016; Bruce-Keller et al., 2017; Lipton et al., 2017).

Far fewer studies have examined overall diet patterns (Borge et al., 2017). Among 23,020 children in the Norwegian Mother and Child Cohort Study, Jacka et al. (2013) reported that “unhealthy” maternal diet patterns during pregnancy were associated with increased externalizing child behaviors. A recent study in the United Kingdom suggested that maternal diets characterized by higher intakes of fruits and vegetables and lower intakes of meat and potatoes are associated with higher child IQ at age 8 years (Freitas-Vilela et al., 2017). In the Generation R study involving 3,104 children, investigators reported decreased levels of externalizing behaviors from children born to mothers who adhered to a Mediterranean diet during pregnancy, and increased child externalizing behaviors in children born to mothers who consumed a “Traditionally Dutch” diet consisting of high meat intake (processed and unprocessed), margarines and potatoes (Steenweg-de Graaff et al., 2014).

Precise definitions for the “Mediterranean diet” vary, but all share the following similarities: rich in fresh fruits, vegetables, legumes; higher amounts of mono-unsaturated fatty acids (MUFAs) such as olive oil, whole grains, and fish, while sparse in meat (Trichopoulou et al., 2003; Bach et al., 2006; Sofi, 2009; Korre et al., 2014). The Mediterranean diet has long been associated with a myriad of positive outcomes, both observationally and in randomized controlled trials. These include improved cognition in adults (Hardman et al., 2016), as well as reductions in cancer mortality (Trichopoulou et al., 2003; Schwingshackl et al., 2011; Dilis et al., 2012), all-cause mortality (Trichopoulou et al., 2003; Knoops et al., 2004; Panagiotakos et al., 2006), cardiovascular/chronic heart disease (CVD/CHD) mortality and morbidity (Renaud et al., 1995; Estruch et al., 2006; Martínez-González et al., 2008; Fung et al., 2009; Sofi, 2009; Kastorini et al., 2011), decreased incidence of diabetes mellitus (Estruch et al., 2006; Martínez-González et al., 2008; Esposito et al., 2009), as well as decreases in obesity related metrics (Iris Shai et al., 2008; Esposito et al., 2009; Leighton et al., 2009; Sofi, 2009; Verweij et al., 2011; Korre et al., 2014), and improvement of components of metabolic syndrome (Esposito et al., 2009; Leighton et al., 2009; Kastorini et al., 2011; Korre et al., 2014). In children, Mediterranean diet adherence (MDA) has been associated with reduced odds of ADHD and improved school performance (Esteban-Cornejo et al., 2016; Rios-Hernandez et al., 2017).

Although mechanisms linking Mediterranean diet and outcomes are still unclear, epigenetic modifications are hypothesized as one mechanism to explain associations between environmental cues such as maternal diet, and child temperament/behavior (Harper, 2005; Lee, 2015; Arpon et al., 2016; Lorite Mingot et al., 2017); for review see (Lillycrop and Burdge, 2015). Animal studies have demonstrated changes in DNA methylation in the offspring of mice from dams fed different diets during pregnancy (Vucetic et al., 2010). In addition, dietary choline deficiency in dams has been shown to alter methylation patterns in mouse fetal brains (Niculescu et al., 2006). In humans, low maternal MDA has recently been associated with altered CpG methylation of the imprinted *MEG3-*IG control region in female offspring (Gonzalez-Nahm et al., 2017). In other studies, imprinted *IGF2* was altered at birth in offspring with periconceptional exposure to famine during world war II (1944–1945) (Tobi et al., 2009). Additional support for epigenetics as a mechanism for modulating behavior in humans is recent work identifying altered methylation in brain associated with schizophrenia and bipolar disorders (Xiao et al., 2014), altered cord-blood methylation patterns in *DRD4* and *5-HTT* associated with ADHD symptoms in children (van Mil et al., 2014), and work by Fuemmeler et al. (2016) relating methylation of the regulatory regions of imprinted genes with infant temperament.

Sex-specific methylation differences are common in the genome (Singmann et al., 2015). As shown in Gonzalez-Nahm’s work (Gonzalez-Nahm et al., 2017), differential methylation of imprinted genes from maternal dietary exposures in offspring can also be sex-specific. Additional evidence for sex-specific differences in the methylation of imprinted control regions (ICRs) in response to prenatal exposures comes from work showing that female infants, but not males, exhibited differential methylation of ICRs of *IGF2* and *H19* in response to maternal anxiety (Mansell et al., 2016). Likewise, lead exposures have been associated with sex-specific changes to the methylation patterns in multiple ICRs (Goodrich et al., 2015; Li Y. et al., 2016), and while not statistically significant, sex-specific differential methylation of the *H19* and *IFG2* DMRs were associated with prenatal exposure to cigarette smoke (Murphy et al., 2012a).

In the current study, we sought to test the hypothesis that maternal adherence to a dietary pattern favorably associated with multiple health outcomes (Mediterranean) would be associated with offspring behavior and changes in methylation of ICRs, many of which have been associated with other behavioral offspring outcomes from prenatal exposures.

## Materials and Methods

### NEST Cohort and Study Population

Study participants included mother and infant dyads enrolled in the Newborn Epigenetics STudy (NEST), a prospective cohort for which accrual protocols have been previously described in detail (Liu et al., 2012). The target population was first trimester pregnant women visiting prenatal clinics of Duke Hospital and Duke affiliated clinics. To be included, women had to be aged 18 years and older and speak English or Spanish. From these, women with an established HIV infection or who intended to relinquish custody of offspring were excluded. To facilitate specimen collection at birth, women who receive obstetrics care at hospitals other than Duke Obstetrics or Durham Regional Hospitals were also excluded. Between 2009 and 2011, 1700 pregnant women were enrolled, and 396 were lost to the cohort due to fetal wastage (*n* = 113), refusal of further participation (*n* = 146), received obstetric care at an outside hospital (*n* = 114), or other reasons (*n* = 23), such that 1,304 (76.7%) women remained enrolled in the study up to the time of analysis. The recruitment protocol was approved by the Duke University Institutional Review Board.

These analyses are limited to 325 mother child pairs (excluding multiple births) from which mothers completed both a periconceptional-food frequency questionnaire (FFQ) (University of Texas, MD Anderson Cancer Center Nutrition and Lifestyle Core Questionnaire 2008v.2) either upon enrollment or in their first trimester, and an Infant Toddler Social and Emotional Assessment (ITSEA) during the child’s second year of life. Because we were interested in maternal diet and because of the potential epigenetic vulnerability due to the requirement to maintain methylation at imprinted DMRs during the reprogramming that occurs immediately post-fertilization, mothers were specifically asked to recall foods consumed at or near their last menstrual cycle. FFQ responses were converted to intakes of foods, food groups, energy, and nutrients by Nutrition Quest^1^.

### Child Behavioral Outcomes

The ITSEA (Carter et al., 2003) was administered by a parent, caregiver or staff to children aged 12–24 months (mean = 13.9 months) during a NEST follow-up visit. The ITSEA tool has been validated and used extensively to examine social-emotional behavioral outcomes and temperament in early-childhood (Carter et al., 2003, 2010). Scales of the ITSEA have demonstrated acceptable test–retest reliability and inter-rater reliability; Cronbach’s alpha coefficients for the internalizing and externalizing domains were 0.80 and 0.86, respectively (Carter et al., 2010). The ITSEA consists of questions regarding *Externalizing* behaviors (with domains for *aggression-defiance*, *peer-aggression*, and *impulsive-activity*), *Internalizing* behaviors (*anxiety, separation-distress, depression-withdrawal*, and *inhibition-to-novelty*), *Dysregulation* related behaviors (*sleep, negative-emotionality, eating*, and *sensory-sensitivity*), and *Competency* (*attention, compliance, imitation/play, mastery-motivation*, and *empathy*). In addition, the ITSEA contains 13 questions to assess *Maladaptive* behavior, 10 questions to assess *Social-Relatedness* behavior and 8 questions to assess *Atypical* behavior. We also examined a composite autism spectrum disorder index calculated from the problem and competency portions of the ITSEA as described in Kruizinga et al. (2014). With the exception of the *Social-Relatedness* category and all *Competency* domains, increased scores indicate adverse behavioral outcomes.

**FIGURE 1:**
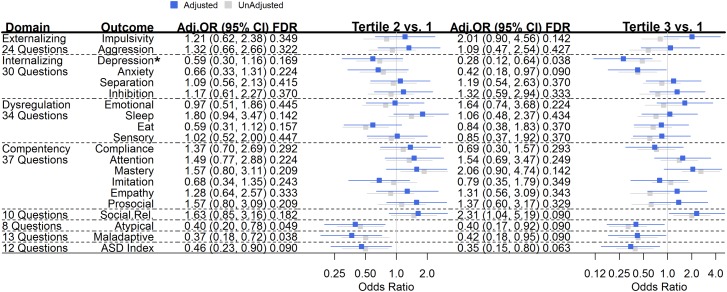
Maternal Mediterranean Diet Adherence (MDA) and Child Behavior Outcomes. For a given tertile of maternal MDA compared to tertile 1 (referent), the odds ratio (95% confidence interval) represents the risk of being in a higher tertile of behavioral outcome. Unadjusted (gray) and adjusted (blue) odds ratios (95% confidence intervals) are plotted. Estimates were adjusted for breastfeeding at least 3 months, age of child at behavioral assessment, maternal fiber intake, total calories, folate, education, diabetes, obesity, smoking, and age, as well as paternal age and child parity, premature birth, weight, race, and child sex. *Q-*values are shown and represent the false discovery rate (FDR) for each finding, which is automatically corrected for multiple testing. ^∗^Bonferroni-corrected *p*-trend = 0.041.

Infant Toddler Social and Emotional Assessment questions are scored from 0 to 2 (0 = Not True/Rarely, 1 = Somewhat, 2 = Very True/Often) and final domains are scaled by the number of questions in the domain. To examine associations of diet on ITSEA assessed child behaviors (**Figure 1**), and as many items were not normally distributed, even after log-transformation (**Supplementary Figure 1**), ITSEA behavior scores were divided into tertiles and associations with dietary exposures were assessed using ordinal logistic regression. We did not assess the *peer aggression* subscale of externalizing behavior due to lack of non-zero scoring (>2/3 of data were zero; data not shown).

### Mediterranean Diet Scoring

As described in detail by Gonzalez-Nahm et al. (2017), pregnant women’s MDA was calculated from the food frequency questionnaire (FFQ) upon enrollment soon after conception, concerning dietary consumption at or near their last menstrual period. In brief, intakes of food categories were adjusted by total energy to grams/1000 kcals and a modified version of Trichopoulou’s Mediterranean diet scoring was used (Trichopoulou et al., 2003). Participants were scored a 0 for below-median consumption of meats (including red meat, pork, poultry, game, but excluding processed meats) and a 1 for above-median consumption of the following: fruit (including fresh, dried and frozen, but excluding juice), vegetables (excluding vegetable juice and white potatoes), fish, dairy (including full-fat dairy but excluding dairy desserts), whole grains, nuts and seeds (including nut-butters), beans and legumes (including soy), and the ratio of mono-unsaturated fat to saturated fat. Alcohol consumption was not scored as it is not recommended during pregnancy and was extremely low in this cohort. The final Mediterranean diet score ranged from 0 to 9 with 9 as most adherent (**Supplementary Figure 2**). This composite MDA score was divided into tertiles (referent = first tertile) and subsequently used to assess associations of MDA with child behaviors. The normal distribution of these scores (**Supplementary Figure 2**) was maintained by offspring sex and by the sub-cohort used to assess associations of maternal MDA on CpG methylation.

### DNA Methylation Assessment

For *n* = 142 children of the 325 included, 800 ng of genomic DNA from umbilical cord-blood was treated with sodium bisulfite. Treated DNA (40 ng) was used for bisulfite pyrosequencing (Fuemmeler et al., 2016; Gonzalez-Nahm et al., 2017) which included 48 CpG sites across 8 differentially methylated regions (DMRs) that regulate one more of nine imprinted genes [*IGF2*, *H19*, *MEG3*, *MEG3-*IG (regulates *MEG3 and DKL1)*, *NNAT*, *MEST*, *SGCE/PEG10*, and *PLAG1*]. These regions are described in detail elsewhere (Fuemmeler et al., 2016; Cowley et al., 2018), as are complete assay conditions including primers and post-assay quality control (Murphy et al., 2012b; Nye et al., 2013; Gonzalez-Nahm et al., 2017). For the assessment of MDA on methylation, the number of subjects with maternal dietary information, all covariates, and cord blood methylation varied.

### Statistical Analysis

For analysis of child behavioral outcomes, the following maternal covariates were included: age at delivery, education level (any college vs. none) pre-pregnancy obesity (BMI > 30 vs. BMI < 30), race (white, black, Hispanic, other), smoking during pregnancy, any of gestational, type I or type II diabetes, daily intake of folate (diet plus supplements: <400 μg, 400–800 μg, >800 μg), daily fiber intake, daily energy intake, and any breastfeeding of at least 3 months. We also adjusted for paternal age as well as child sex, child birthweight, full term status (≥37 weeks gestation or not), parity (nulliparous vs. not), and age of ITSEA assessment (**Table 1**). As neither post-birth-breastfeeding-status nor child age at ITSEA would be expected to impact cord-blood methylation patterns, these covariates were removed for assessment of associations of diet with differentially methylated imprinted gene DMR CpGs in cord-blood.

**Table 1:** Study demographics.

	Current Study(*N* = 325)	
**Categorical Covariates**	***N***	**%**
Child sex		
Male	174	53.5
Female	151	46.5
Child race		
White	138	42.5
Black	95	29.2
Hispanic	72	22.2
Other	20	6.2
Maternal education		
No college	119	37.5
Some college	198	62.5
Maternal smoking		
No	283	88.7
Yes	36	11.3
Maternal obesity		
BMI < 30	244	75.5
BMI ≥ 30	79	24.5
Child preterm birth		
Gestation ≥ 37w	296	91.9
Gestation < 37w	26	8.1
Diabetes (Any)		
No	286	91.7
Yes	26	8.3
Child parity		
Nulliparous (0)	142	43.7
Multiparous (≥1)	183	56.3
Breastfeeding		
<3 months	99 232	30.5
≥3 months	226	69.5
**Continuous Covariates**	**Mean**	***SD***
Maternal fiber (g)	30.3	16.6
Maternal energy (kcal)	2598	1357
Maternal age (y)	29.1	5.5
Paternal age (y)	30.8	6.5
Daily folate (total)(μg)	666.3	364.0
Child weight (g)	3318	564
Child assessment age (m)	13.9	2.37


All analyses were done using *R* version 3.4.2 (R Development Core Team. R Foundation for Statistical Computing, Vienna, Austria). For associations of maternal diet (tertiles) with child behavior (tertiles), ordinal logistic regression was conducted using polr() from the *MASS* package (Venables and Ripley, 2002). Trend tests were conducted with ordinal logistic regression on tertiles of behavioral outcome while treating tertiles of maternal MDA exposure as a linear predictor (1 = least adherent, 3 = most adherent). In results, unless otherwise specified, trend *p*-values are not adjusted. For assessing the relationship between maternal dietary adherence and the percent methylation of CpGs (continuous) as well as the relationship between percent methylation of CpGs and child behavior (continuous), linear regression was conducted using lm() from the base R stats package. Correction for multiple comparisons was performed by (i) control of the family-wise error rate (FWER) at α= 0.05, and (ii) control of the false-discovery rate (FDR) at level 0.15. The use of FWER control is intended to highlight findings that pass the most rigorous false-positive standards, while the FDR procedure allows a proportion of false positives while maintaining a high proportion (0.85) of true discoveries. FWER control was achieved by applying the Bonferroni correction, augmented by permutation analysis for each set of tests. The permutation analyses used covariate-residualized of responses, permuted relative to the covariate-residualized predictors of interest. An alternate analysis used 10,000 permutations of DMR β values vs. maternal MDA, covariate-residualized and using the partial correlations as the test statistic. Control of the FWER over all DMRs was performed by using the minimum *p*-values over DMR as a statistic. In all instances, the permutation-adjusted *p*-values were only slightly more significant than the Bonferroni-corrected values, and did not result in additional significant findings, and so the Bonferroni-corrected values are reported for simplicity. FDR *q*-values were computed using the Bioconductor *qvalue* package (Storey et al., 2015), version 2.10.0 using default settings and are, by construction, already multiple comparison corrected.

## Results

### Cohort Demographics Outcome Assessment

Approximately half of the children assessed were male (53.5%) (**Table 1**). The majority of mothers identified as white (42.5%), with 29.2% as black, 22.2% as Hispanic, and the remainder (6.2%) classified as other. Nearly all births were full-term (91.9%) and 43.7% of children were born to nulliparous women. Amongst mothers, 62.5% had some college, 11.3% smoked while pregnant, and 24.5% were classified as obese (BMI ≥ 30) at or near conception. The prevalence of any of gestational, type I or type II diabetes was 8.3% (**Table 1**). Nearly 70% of mothers reported breastfeeding for at least 3 months. Children were between the age of 12 and 24 months when assessed for behavior (average age, 13.9 months). The distributions and medians for assessed behavioral outcomes are summarized in **Supplementary Figure 1**. With the exception of *social relatedness* index and the *Competency* subscales (*compliance, attention, mastery, imitation, empathy, and prosocial*), lower scores indicate favorable behavior outcomes.

### Maternal Mediterranean Diet and Child Behavior Outcomes

We examined associations between maternal MDA at or near conception with ITSEA assessed childhood behaviors in offspring 12 and 24 months of age. The distribution of maternal MDA is shown in **Supplementary Figure 2**. Maternal MDA scores were divided into tertiles with tertile 1 (referent) as the least adherent.

Unadjusted and adjusted odds ratios for associations of maternal MDA and offspring behaviors assessed in the second year of age are displayed in **Figure 1**. Amongst *Internalizing* behaviors, when comparing offspring born to women with the lowest tertile of MDA, offspring of mothers in the highest tertile of MDA were less likely to score in a higher tertile of *depression* [OR (95% CI) = 0.28 (0.12, 0.64); *p*-trend = 0.002, Bonferroni-corrected *p*-trend = 0.041] and *anxiety* [0.42 (0.18, 0.97); *p*-trend = 0.041]. While not statistically significant, these associations persisted when comparing offspring of mothers in the middle tertile of MDA compared to those with mothers in the lowest level of MDA [*depression*: 0.59 (0.30, 1.16); *anxiety*: 0.66 (0.33, 1.31)]. The highest tertile of maternal MDA exposure was also associated with improved *social relatedness* behaviors [2.31 (1.04, 51.9); *p*-trend = 0.041] in offspring. Maternal MDA was not associated with *Externalizing*, *Dysregulation* or *Competency* behaviors (**Figure 1**).

When compared to offspring born to women with MDA scores in the lowest tertile, offspring of women with MDA scores in the middle tertile were less likely to report *atypical* behaviors [OR (95% CI) = (0.40, (0.20, 0.78)); associations of similar magnitude persisted when the highest tertile was compared to the lowest tertile (0.40 (0.17, 0.92); *p*-trend = 0.049]. Further, maternal MDA was inversely associated with *maladaptive* behaviors [middle tertile MDA vs. low – 0.37 (0.18, 0.72); high tertile MDA vs. low – 0.42, (0.18, 0.95); *p*-trend = 0.077]. Lastly, when compared with offspring of women with low MDA, offspring of women with middle and high scores of MDA were less likely to score in a higher tertile of *autism spectrum* index behaviors [middle tertile vs. low – 0.46, (0.23, 0.90); high tertile vs. low – 0.35 (0.15, 0.80); *p*-trend = 0.017] (**Figure 1**).

Child behavior can vary by sex; we examined if associations between maternal MDA and offspring behaviors did so (**Supplementary Figure 3**). In males, offspring born to mothers with the highest MDA scores were more likely to score in the next highest tertile of *social relatedness* behaviors [OR (95% CI) 3.20 (1.04, 10.25; *p*-trend = 0.032)]. Inverse associations of maternal MDA on *depression, maladaptive*, and *ASD index* behaviors were pronounced in females. Compared with daughters born to mothers with the lowest MDA scores, daughters born to mothers with MDA scores in the middle and high tertiles were much less likely to exhibit *depression* behaviors [middle tertile vs. low: 0.22 (0.06, 0.69); highest tertile vs. low: 0.05 (0.01, 0.22); Bonferroni-corrected *p*-trend = 0.002], or *maladaptive* behaviors [middle tertile vs. low: 0.23 (0.08, 0.63); highest tertile vs. low: 0.20 (0.05, 0.77); *p*-trend = 0.031] or *ASD*-related behaviors [middle tertile vs. low: 0.24 (0.08, 0.72); highest tertile vs. low: 0.08 (0.02, 0.38); Bonferroni-corrected *p*-trend = 0.017]. Females born to women in the highest tertile of MDA scores were more likely to exhibit increased *mastery* [5.53 (1.44, 22.2); *p*-trend = 0.012] and *empathy* [5.74 (1.36, 26.2); *p*-trend = 0.015] behaviors when compared with mothers in the lowest tertile of MDA scores.

### Maternal MDA and CpG Methylation in Offspring

**FIGURE 2:**
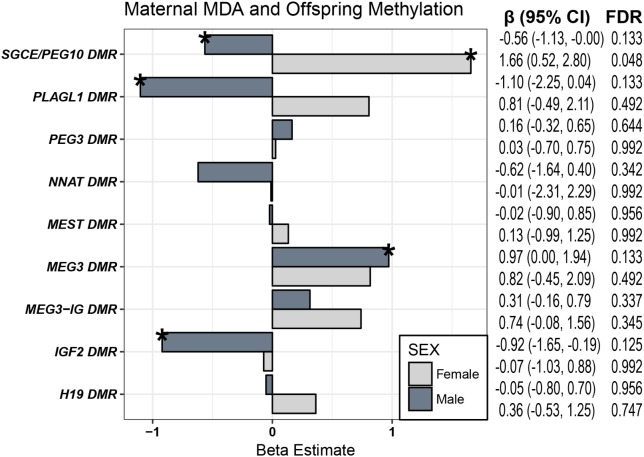
Maternal MDA and CpG Methylation. Females (light gray) and males (dark gray) were evaluated separately with linear regression for associations of maternal MDA on the average methylation status of the control region of 9 imprinted gene loci. Effect estimates for mean CpG methylation were adjusted for breastfeeding at least 3 months, age of child at behavioral assessment, maternal fiber intake, total calories, folate, education, diabetes, obesity, smoking, and age, as well as paternal age and child parity, premature birth, weight, race (^∗^FDR *q* < 0.15; *n* ranged from 51 to 75).

To test the hypothesis that associations of maternal MDA on offspring behavior are mediated through epigenetic mechanisms and taking into consideration work linking differential methylation control regions of imprinted genes with both offspring behavioral outcomes (Fuemmeler et al., 2016), and maternal diet (Rijlaarsdam et al., 2017), we examined the relationship of diet on CpG methylation of DMRs regulating nine imprinted genes. Building on prior research in our group on imprinted genes, we assessed the methylation status of 48 CpGs in the regulatory DMRs of nine imprinted genes for a subset of the cohort (*n* = 142 mother/child pairs). These include the DMRs of *PLAGL1, H19*, *SGCE/PEG10, PEG3, NNAT, MEST*, and two DMRs each for the *DLK1/MEG3* and *IGF2* domains. As mentioned in the introduction, since methylation of ICRs often vary by sex, we stratified on sex for these analyses and assessed whether maternal MDA was associated with the mean methylation percentage of CpGs at these loci with covariate-adjusted multiple linear regression (**Figure 2**). In females, maternal MDA was associated with an increase in mean methylation of the ICR of *SGCD/PEG10* [β (95% CI) = 1.65 (0.52, 2.80); Bonferroni-corrected *p*-value = 0.048; **Figure 2**]. This translates to a 1.65% increase in mean methylation of the control region of *IGF2* in offspring cord-blood for each unit increase in maternal MDA from zero to nine (**Supplementary Figure 2**). In males, after adjusting for a false discovery rate (FDR) of 0.15, maternal MDA was associated with a decrease in the mean methylation of CpGs in the *IGF2* DMR [β (95% CI) = -0.92 (-1.65, -0.19); **Figure 2**] as well as with decreased methylation of the *SGCE/PEG10* [-0.56 (-1.13, -0.00)], and *PLAGL1* [-1.10 (-2.25, 0.04)] DMRs, and an increase in the mean methylation at the *MEG3* DMR [β (95% CI) = 0.97 (0.00–1.97)]. For each of these findings, the effect estimates across the interrogated DMR were consistent in magnitude and direction (**Supplementary Figure 4**). Further, while not statistically significant in females, increased maternal MDA was consistently associated across the interrogated DMRs with increased methylation of CpGs in the *MEG3* DMR, the intergenic *MEG3-*IG DMR and the *PLAGL1* DMR (**Supplementary Table 1** and **Supplementary Figure 4**).

### CpG Methylation and Child Behavior

**FIGURE 3:**
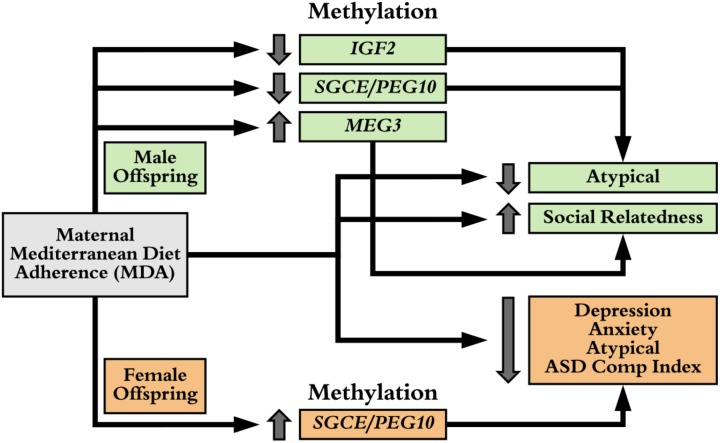
Sex Specific Maternal MDA and CpG Associations on Child Behavior. In females (orange), maternal MDA is associated with increased methylation in the control region of SGCE/PEG10 locus. In turn, this is associated with decreased odds of depression, anxiety, atypical, and Autism Spectrum Disorder (ASD) composite index behaviors, as is maternal MDA. In males (green), maternal MDA is associated with hypo-methylation of the control region of IGF2 and SGCE/PEG10 loci and hyper-methylation of the MEG3 control region which are, in turn, are consistently associated with maternal MDA associations on atypical and social relatedness behaviors.

For the significant associations of maternal MDA on offspring behavior (**Figure 1**) and maternal MDA on methylation of imprinted gene control regions (**Figure 2**), we examined associations of mean methylation on child behaviors (**Supplementary Figure 5**). In males, increased methylation of *IGF2* was associated with increased *ASD Composite Index* behaviors and increased *Atypical* behavior, increased methylation of *MEG3* was associated with increased *Social Relatedness* and decreased *Maladaptive* behaviors, increased methylation of *SGCE/PEG10* was associated with increased *Atypical* behavior, while increased methylation of the *PLAGL1* DMR was associated with increased *Atypical* and *ASD Composite Index* behaviors (**Supplementary Figure 5**). In females, increased methylation of the control region of *SGCE/PEG10* was associated with decreased risk of *depression, anxiety*, and *atypical* behaviors. These estimates were unstable as evidenced by large confidence bounds and we were underpowered to conduct mediation analyses. In spite of this, we were able to identify multiple consistent associations of maternal MDA on child behavior, of maternal MDA on differential methylation of imprinted gene control regions, and of these DMRs on child behavior (**Figure 3**).

## Discussion

While much research has been conducted on the role of individual nutrients or foods on child outcomes, few studies have examined the potential role of the overall quality of maternal diet during pregnancy on child behavioral and other neurodevelopmental outcomes (Borge et al., 2017); the results so far are limited. In this study of a subset of the ethnically diverse NEST cohort, we observed a reduced risk of adverse child behaviors in relation to maternal diet patterns around the time of conception. Maternal MDA was associated with child social relatedness behaviors, and inversely associated with the internalization subscales of depression and anxiety. Further, maternal MDA was inversely associated with atypical, maladaptive, and ASD behavioral patterns. To minimize maternal depression as a potential confounder of the association between maternal MDA and offspring *depression* (**Figure 1**), we included maternal post-natal depression in our adjusted model and estimates were materially unchanged (data not shown). In addition, although covariate proportions differed (except sex and child birthweight), between our cohort and the parent cohort (data not shown), estimates for our significant findings were materially unchanged between unadjusted and adjusted models (**Figure 1**).

Our results suggest that during pregnancy, maternal adherence to the Mediterranean Diet, rich in legumes, vegetables, fish, and healthy fatty acids, and lower in red meat, may promote behavioral and emotional well-being in children. These findings are consistent with earlier studies reporting that maternal MDA, or to a dietary principal components pattern characterized as “healthy,” is beneficially associated with child behavioral and emotional problems (Jacka et al., 2013; Steenweg-de Graaff et al., 2014). In adults, adherence to this diet pattern is associated with better cognitive outcomes and decreased depression, suggesting a role of diet on cognition and emotional functioning (Crichton et al., 2013; Rienks et al., 2013; Trovato et al., 2014; Hardman et al., 2016; Aridi et al., 2017; Loughrey et al., 2017; Molendijk et al., 2018). Maternal adherence to this diet pattern during pregnancy has also been associated with protection from allergic disease in human offspring (Netting et al., 2014; Castro-Rodriguez and Garcia-Marcos, 2017). Given the rapid increase in the incidence of neurodevelopmental disorders in children (CDC, 2010; Visser et al., 2013, 2014), if replicated, our results relating maternal MDA during gestation with child neurodevelopmental outcomes may present new avenues for prevention.

Consistent with our findings, laboratory studies suggest that maternal diet during pregnancy may affect offspring behavioral health. Numerous studies in rodents have found associations between maternal diet and offspring neurodevelopment and behavior, such as recent work associating high maternal caloric intake with increased anxiety in male rats (Balsevich et al., 2016). Offspring from female rats fed a high fat diet pre-gestational through lactation suffered cognitive deficits that were ameliorated by dams who also had access to exercise (Moser et al., 2017). Anxiety, cognition, and compulsive behaviors were altered in male mice weaned from mothers who had received gut microbiota from high fat diet mice (Bruce-Keller et al., 2017). Rather than reductions in fat or increases in energy intake, the Mediterranean diet is thought to beneficially influence health outcomes as a result of increases in intakes of micronutrients, including MUFAs, antioxidant vitamins, and phytochemicals (Ros et al., 2014). Most rodent research to date has focused on the *high-fat* paradigm when examining outcomes. Further, nearly all studies examine high-fat in the context of excess energy, further reducing the ability to elucidate whether observed changes in offspring are due to excess body weight, excess energy or fat intake, or some combination.

Imprinted genes have parental allele specific silencing via epigenetics. These patterns are laid down during gametogenesis in imprinting control regions (Bartolomei and Ferguson-Smith, 2011). Germline ICRs are typically more epigenetically stable throughout life and aberrant epigenetic marks for imprinted gene ICRs are associated with effects on metabolism, neurodevelopment, and growth. Because of our findings that maternal MDA is associated with changes in methylation patterns of imprinted genes, changes which can vary by sex, we did re-examine associations of maternal MDA on child behavior stratified by sex despite being underpowered to gain real insight. For a subset of our cohort, we were able to conduct targeted assessment of the CpGs in the control regions of imprinted genes (**Supplementary Figure 4**), providing evidence for novel associations of maternal diet with changes in methylation in imprinted ICRs of offspring. Our data suggest that high maternal MDA is associated with altered methylation of the *MEG3* ICR independent of sex, and of the *SGCE/PEG10, PLAGL1* and *IGF2* ICRs in a sex-dependent manner. These changes in methylation, are in turn, consistently associated with offspring behaviors that are also associated with maternal MDA (**Figure 3**).

We report here that maternal MDA is associated with increased methylation of the maternally expressed 3 (*MEG3*) ICR in males. Several lines of evidence suggest that increased methylation of the *MEG3* ICR, regulating a known tumor suppressor (Zhou et al., 2012), is associated with decreased expression of *MEG3*, albeit with increased expression of the reciprocally imprinted *DLK1* gene (Murphy et al., 2006). In genome-wide methylation arrays (Markunas et al., 2014), *MEG3* DMR methylation was associated with maternal smoking and in human adults, *DLK1* dysregulation has been associated with schizophrenia (Gardiner et al., 2012). In both mice and humans, hypo-methylation of the *MEG3* control region has been associated with adverse neurobehavioral phenotypes (Kagami et al., 2015; Fuemmeler et al., 2016; Drobna et al., 2018). Microarray data from the mouse forebrain have shown differential spatial expression of the imprinted gene *Gtl2* (aka *Meg3*) between the ventral and dorsal telencephalon of the mouse at a critical time point in the generation and migration of cortical neuronal populations (McLaughlin et al., 2006). In an effort to boost power, and given the sex independent associations of maternal MDA on the two control regions associated with *MEG3*, we examined if methylation at these loci mediated our findings of associations of maternal MDA on behavior, but were underpowered to draw conclusions (data not shown).

We also found sex-dependent associations of maternal MDA and the methylation of the epsilon sarcoglycan and paternally expressed gene 10 (*SGCE/PEG10*) ICR. *PEG10* is essential for proper placental development, and over-expression of PEG10 protein has been associated with multiple tumor phenotypes (Okabe et al., 2003; Li X. et al., 2016; Peng et al., 2016), while differential methylation of the *SGCE/PEG10* ICR has also been associated with cancer (Sepulveda et al., 2016). Hypomethylation of the *SGCE/PEG10* ICR has been associated with higher expression of *PEG10* as well as maternal stress and depression, although mechanisms are still unclear (Liu et al., 2012). In males at age 1 and 3, increased methylation at *PEG10* has been associated with greater weight for length ratios (Gonzalez-Nahm et al., 2018), and paternal obesity has been associated with decreased methylation of *PEG10* in sperm (Soubry et al., 2016).

Less is known about Pleiomorphic adenoma-like protein 1 (*PLAGL1* or *ZAC1*). *PLAGL1* is a zinc finger protein associated with cell growth suppression and with transient neonatal diabetes mellitus (Kamiya et al., 2000; Varrault et al., 2001). We found maternal MDA associated with hypomethylation of the ICR in males. Interestingly, in the EDEN cohort, maternal alcohol and dietary vitamin B2 were associated with *ZAC1* methylation while increased *ZAC1* methylation was associated with estimated fetal weight, weight at birth and at 1 year of age (Azzi et al., 2014).

Lastly, we report a strong association of maternal MDA with hypomethylation of insulin-like growth factor 2 (*IGF2*) in male offspring as well as an association of *IGF2* hypomethylation with decreased *atypical* behavior (**Figure 3**). Hypomethylation of *IGF2* and increased IGF2 protein have been associated with paternal obesity, as well as increased offspring obesity risk (Lawlor et al., 2012; Tobi et al., 2014; Dunford and Sangster, 2017). Decreased methylation of the *IGF2* DMR has been associated with increased *IGF2* transcripts (Murphy et al., 2012b) and protein (Hoyo et al., 2012). Differential methylation of *IGF2* has been associated with both periconceptual exposure to extreme caloric restriction during the Dutch Famine (Tobi et al., 2009), and lower circulating folate concentrations (Hoyo et al., 2014). In the ALSPAC cohort, prenatal dietary patterns comprising high fat and high sugar were associated with lower *IGF2* methylation and an earlier onset of ADHD behaviors (Rijlaarsdam et al., 2017). Dunford and Sangster provide a more thorough review of parental nutrition and offspring epigenetics in the context of metabolic syndrome risk (Dunford and Sangster, 2017).

Additional studies are needed to replicate these findings, but together, these data support mechanistic relationships between high adherence to Mediterranean diet and shifts in methylation of regulatory sequences of imprinted genes in offspring.

We adjusted for a wide array of covariates, including factors such as maternal education, BMI, maternal energy intake, and breastfeeding. Nonetheless, an important limitation of this research is uncertainty of the extent to which associations between child neurodevelopment and maternal diet quality during pregnancy may reflect differences in caregiving which are difficult to measure, or be influenced by confounding by factors such as maternal IQ (Der et al., 2006; Horta et al., 2015). Our study was limited to child outcomes in infancy; the persistence of effects in later life, and whether lasting effects may depend on childhood diet, remains uncertain. Though results are promising, more research is needed to elucidate whether this relationship is causal, and to identify the pathways through which maternal adherence to Mediterranean Diet may affect child behavior.

Our study also had some limitations including small sample sizes for stratified analyses. Despite this, all of the overall findings of associations of maternal MDA on offspring behavior and maternal MDA on offspring mean DMR methylation have an FDR < 0.15, suggesting these associations warrant further studies to replicate these findings. A lack of dietary data in infants to evaluate the extent to which neurodevelopmental outcomes may have been driven by postnatal dietary exposures other than breastfeeding is a limiting factor of this study, although dietary variability should be somewhat homogeneous at the age of behavioral assessment [12–24 months (mean = 13.9 months)]. Ideally, one would have whole-genome methylation data to identify the strongest associations of diet with CpG methylation. Our study has only focused on the effects at imprinted DMRs; there are likely effects at many other regions throughout the genome that more comprehensive epigenetic analyses would reveal. Although maternal MDA was associated with half of the imprinted gene regions evaluated, many of these imprinted gene control regions are related to major cell proliferation pathways such as *TGF*-β and *TP53* which would impact vast downstream signaling pathways, so this may be expected. Further, we have not examined any potential effects on other epigenetic regulatory elements, including histone modifications or the expression and actions of non-coding RNAs. Additional epigenome-wide studies are needed to both replicate and clarify these findings and to characterize an epigenomic signature associated with maternal diet. Recall bias in dietary recall questionnaires is often an issue. To minimize recall bias, mothers in our study were asked to fill out a FFQ about the foods they ate near conception inside of a dozen weeks.

## Conclusion

We report novel significant associations of maternal periconceptional MDA both with positive neurodevelopmental phenotypes in offspring as well as associations of maternal MDA on differential methylation of CpGs in the control regions of imprinted genes. If confirmed in other studies, these findings may pave the way for early identification of adverse behavior risk in offspring and for tailored interventions.

## Data Availability

The datasets for this manuscript are not publicly available because: Human subjects were used with consent in this study, but public release of data is not approved under the IRB. Requests to access the datasets should be directed to CH for approval.

## Ethics Statement

This study was carried outin accordance with the recommendations of the Duke University Health System Institutional Review Board. It conforms with special protections for pregnant women described in 45 CFR 46, Subpart B. The protocol was approved by the Duke University Health System Institutional Review Board. All subjects gave written informed consent in accordance with the Declaration of Helsinki.

## Author Contributions

JH conceived and conducted the analyses, generated the figures and wrote the manuscript. MM, BF, FW, and CH supported the research and assisted with subject matter expertise, analysis plans, and manuscript writing and editing. RM, SG-N, ZH, JD, and SM each assisted with manuscript preparation, analysis support, and editing.

## Conflict of Interest Statement

The authors declare that the research was conducted in the absence of any commercial or financial relationships that could be construed as a potential conflict of interest.

## Footnotes

1 www.nutritionquest.com
