# Supplementary material for: Periconceptional Maternal Mediterranean Diet Is Associated With Favorable Offspring Behaviors and Altered CpG Methylation of Imprinted Genes
Source: Front Cell Dev Biol. 2018 Sep 7;6:107. doi: 10.3389/fcell.2018.00107 (PMC6137242; doi:10.3389/fcell.2018.00107)
Supplement: Supplementary file 1 [file Presentation_1.PDF]

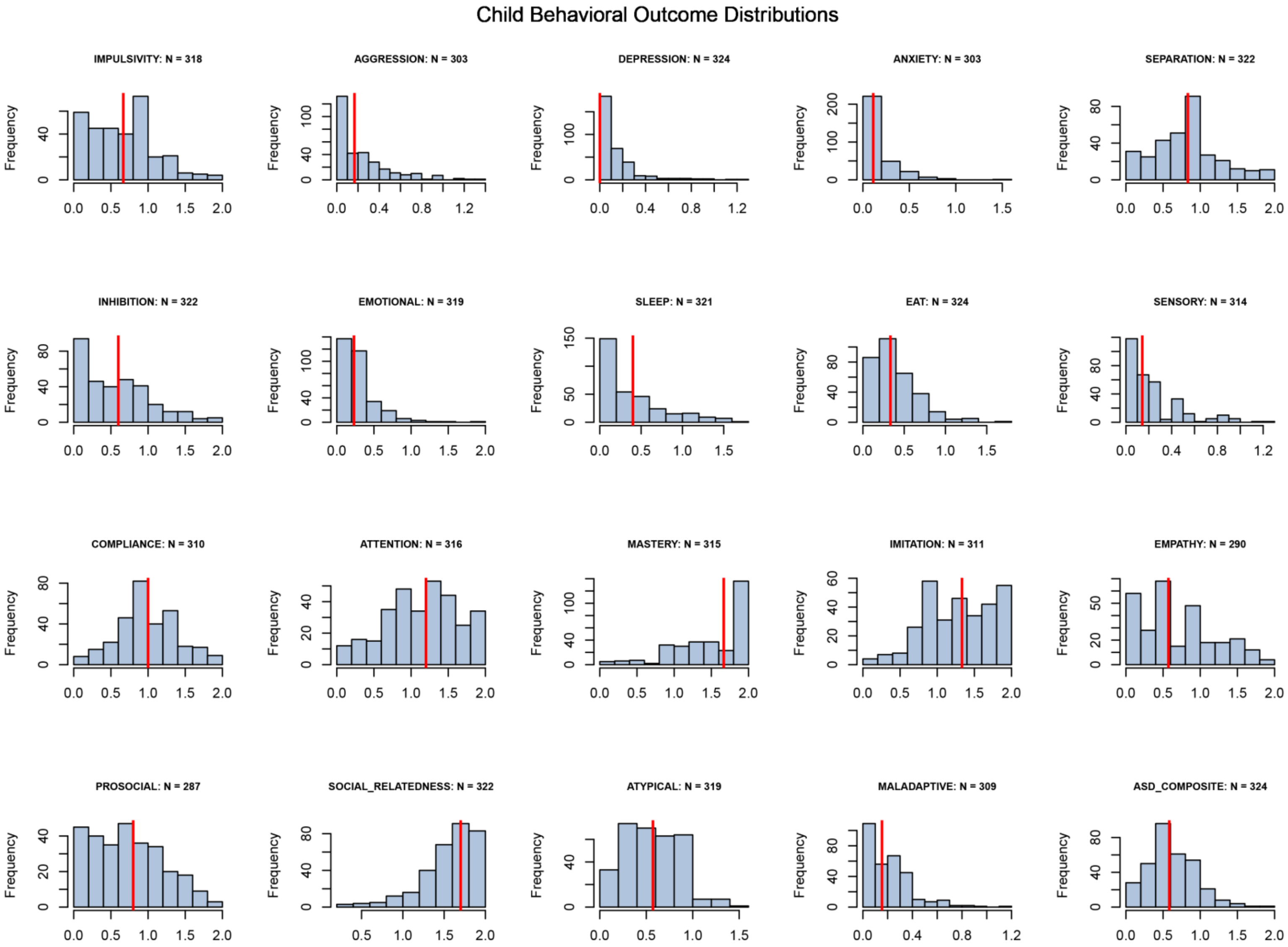

Supplemental Figure 1. ITSEA Behavioral Outcomes. Distributions of child behavioral scores (scaled from 0-2) on the Infant Toddler Social and Emotional Assessment (ITSEA) screen for n = 325 children aged 12-24 months. Higher scores indicate adverse behavioral outcomes in all cases except *compliance, attention, mastery, imitation, empathy, prosocial,* and *social-relatedness* indices. Medians are represented by red lines.

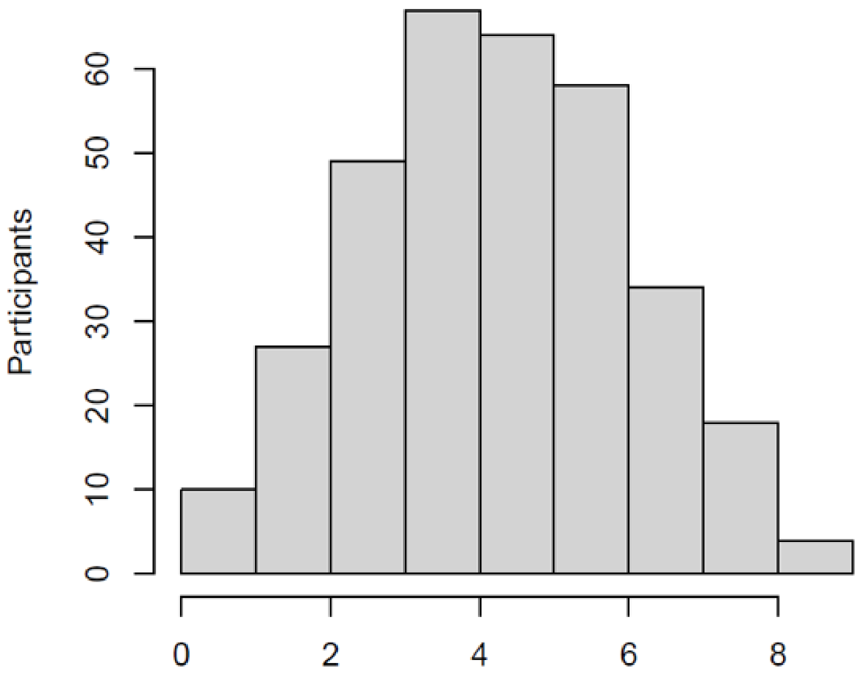

Supplemental Figure 2. Distribution of Maternal Mediterranean Diet Adherence. Participants were scored a 0 for below-median consumption of meats (including red meat, pork, poultry, game, but excluding processed meats) and a 1 for above-median consumption of the following: fruit (including fresh, dried and frozen, but excluding juice), vegetables (excluding vegetable juice and white potatoes), fish, dairy (including full-fat dairy but excluding dairy desserts), whole grains, nuts and seeds (including nut-butters), beans and legumes (including soy), and the ratio of mono-unsaturated fat to saturated fat

Maternal MDA (T2 vs. T1) and Child Behavior by Sex / Maternal MDA (T3 vs. T1) and Child Behavior by Sex

Female ■ Male ■

Outcome	T2 vs. T1 Adj. OR (95% CI) (F)	T2 vs. T1 Adj. OR (95% CI) (M)	T3 vs. T1 Adj. OR (95% CI) (F)	T3 vs. T1 Adj. OR (95% CI) (M)
Impulsivity	2.84 (1.01, 8.25)	0.71 (0.26, 1.90)	4.34 (1.14, 17.46)	1.39 (0.45, 4.33)
Aggression	1.23 (0.44, 3.50)	1.05 (0.37, 2.99)	1.13 (0.32, 4.14)	0.69 (0.19, 2.40)
Depression	0.22 (0.06, 0.69)	1.32 (0.50, 3.53)	0.05 (0.01, 0.22)	0.95 (0.30, 3.03)
Anxiety	0.52 (0.17, 1.52)	0.60 (0.22, 1.60)	0.29 (0.07, 1.16)	0.35 (0.10, 1.12)
Separation	1.31 (0.47, 3.74)	1.07 (0.41, 2.78)	1.13 (0.32, 4.08)	1.32 (0.44, 3.97)
Inhibition	2.28 (0.80, 6.68)	0.73 (0.28, 1.92)	1.29 (0.35, 4.80)	1.27 (0.41, 3.93)
Emotional	1.66 (0.61, 4.64)	0.53 (0.20, 1.38)	2.95 (0.81, 11.24)	0.88 (0.27, 2.82)
Sleep	4.45 (1.62, 12.67)	0.91 (0.35, 2.36)	0.68 (0.19, 2.45)	1.47 (0.46, 4.71)
Eat	0.38 (0.13, 1.03)	0.46 (0.18, 1.21)	0.28 (0.07, 1.02)	1.04 (0.34, 3.20)
Sensory	1.77 (0.60, 5.22)	0.66 (0.24, 1.78)	1.04 (0.27, 3.97)	0.71 (0.22, 2.30)
Compliance	1.15 (0.41, 3.24)	1.54 (0.54, 4.35)	0.99 (0.25, 3.82)	0.44 (0.13, 1.45)
Attention	0.96 (0.35, 2.61)	2.41 (0.92, 6.50)	0.85 (0.23, 3.11)	2.60 (0.82, 8.51)
Mastery	2.21 (0.76, 6.66)	0.88 (0.32, 2.39)	5.53 (1.44, 22.23)	0.70 (0.20, 2.31)
Imitation	0.61 (0.21, 1.77)	0.69 (0.25, 1.87)	0.85 (0.23, 3.16)	0.60 (0.18, 1.92)
Empathy	1.68 (0.54, 5.39)	1.10 (0.40, 3.04)	5.74 (1.36, 26.24)	0.40 (0.11, 1.37)
Prosocial	1.69 (0.59, 4.91)	1.73 (0.62, 4.85)	1.26 (0.33, 4.93)	1.28 (0.37, 4.46)
Social Relatedness	1.57 (0.57, 4.42)	1.40 (0.53, 3.74)	1.53 (0.40, 5.86)	3.20 (1.04, 10.25)
Atypical	0.36 (0.12, 1.05)	0.33 (0.12, 0.90)	0.18 (0.04, 0.73)	0.50 (0.16, 1.58)
Maladaptive	0.23 (0.08, 0.63)	0.65 (0.24, 1.77)	0.20 (0.05, 0.77)	0.78 (0.24, 2.52)
ASD Composite Index	0.24 (0.08, 0.72)	0.81 (0.30, 2.18)	0.08 (0.02, 0.38)	0.70 (0.22, 2.24)

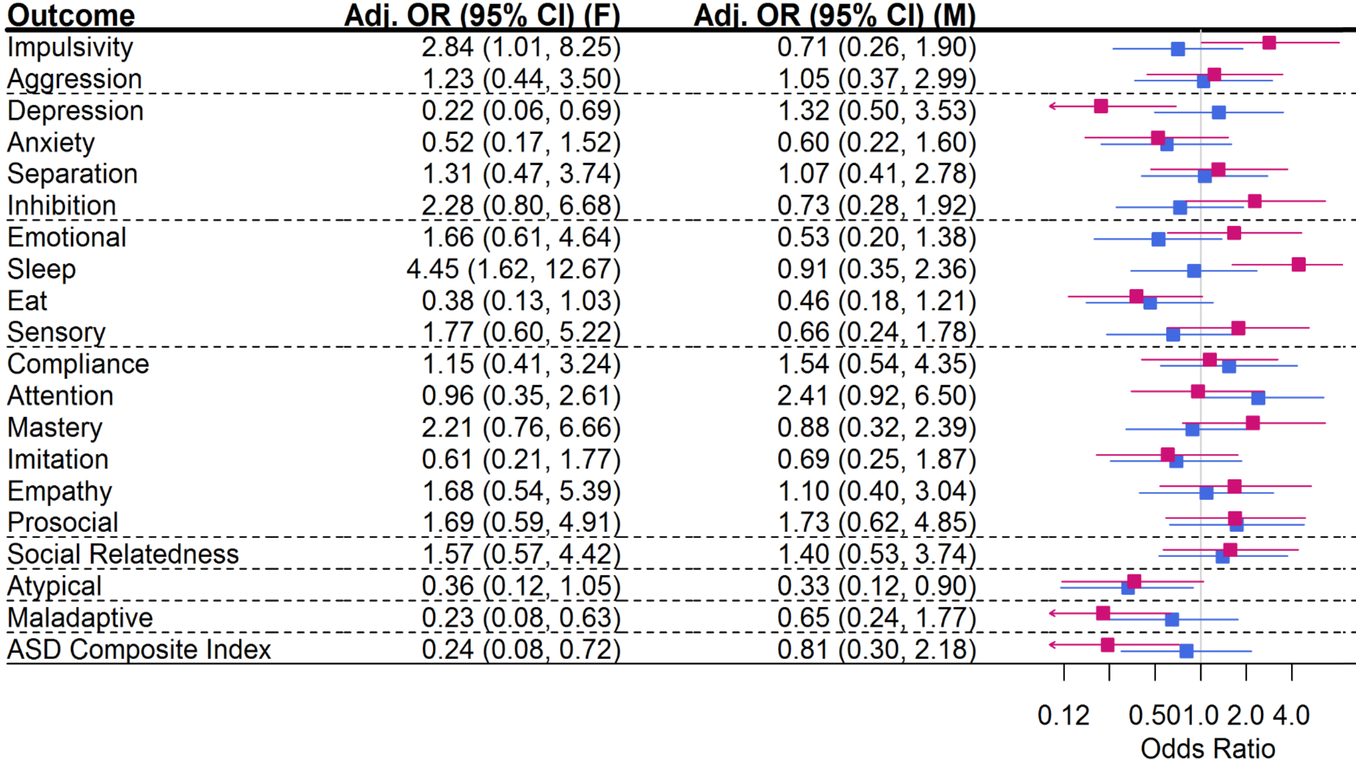

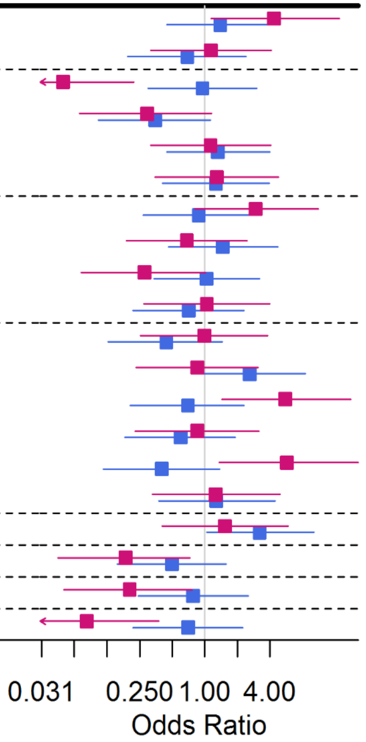

Supplemental Figure 3. Maternal Mediterranean Diet Adherence and Child Behavior Outcomes by Sex. For a given tertile of maternal MDA compared to tertile 1 (referent), the odds ratio (95% confidence interval) represents the risk of being in a higher tertile of behavioral outcome. Males and females were run separately. Female (red) and male (blue) odds ratios (95% confidence intervals) are plotted. Estimates were adjusted for breastfeeding at least 3 months, age of child at behavioral assessment, maternal fiber intake, total calories, folate, education, diabetes, obesity, smoking, and age, as well as paternal age and child parity, premature birth, weight, and race.

CPG/DMR	Females	Males	CPG/DMR	Females	Males
H19_pos1	0.69 (-0.30, 1.68)	-0.14 (-0.95, 0.67)	NNAT_pos1	-0.22 (-2.61, 2.17)	-0.76 (-1.97, 0.46)
H19_pos2	0.32 (-0.62, 1.26)	-0.11 (-0.86, 0.65)	NNAT_pos2	0.32 (-2.18, 2.82)	-0.51 (-1.52, 0.51)
H19_pos3	0.10 (-0.77, 0.97)	0.09 (-0.70, 0.88)	NNAT_pos3	-0.14 (-2.42, 2.14)	-0.60 (-1.62, 0.42)
H19_pos4	0.34 (-0.56, 1.24)	-0.06 (-0.86, 0.75)	NNAT_mean	-0.01 (-2.31, 2.29)	-0.62 (-1.64, 0.40)
H19_mean	0.36 (-0.53, 1.25)	-0.05 (-0.80, 0.70)	PEG3_pos1	0.29 (-0.39, 0.98)	0.23 (-0.27, 0.74)
IGF2_pos1	0.12 (-0.92, 1.16)	-1.07 (-1.93, -0.21)	PEG3_pos2	-0.03 (-0.83, 0.78)	0.39 (-0.32, 1.09)
IGF2_pos2	0.15 (-0.86, 1.17)	-0.97 (-1.75, -0.18)	PEG3_pos3	-0.25 (-1.07, 0.58)	0.06 (-0.61, 0.73)
IGF2_pos3	-0.49 (-1.61, 0.62)	-0.73 (-1.54, 0.08)	PEG3_pos4	-0.02 (-0.78, 0.74)	0.06 (-0.49, 0.61)
IGF2_mean	-0.07 (-1.03, 0.88)	-0.92 (-1.65, -0.19)	PEG3_pos5	0.14 (-0.70, 0.98)	0.19 (-0.44, 0.81)
MEG3_pos1	0.87 (-0.44, 2.17)	0.68 (-0.42, 1.77)	PEG3_pos6	0.00 (-0.79, 0.80)	0.07 (-0.54, 0.68)
MEG3_pos2	1.13 (-0.77, 3.03)	1.35 (0.02, 2.69)	PEG3_pos7	0.10 (-0.62, 0.82)	0.24 (-0.56, 1.05)
MEG3_pos3	0.63 (-0.92, 2.17)	0.88 (-0.22, 1.99)	PEG3_pos8	-0.06 (-0.85, 0.73)	0.27 (-0.22, 0.75)
MEG3_pos4	1.42 (-0.30, 3.14)	1.35 (0.13, 2.56)	PEG3_pos9	-0.02 (-0.84, 0.80)	-0.05 (-0.63, 0.53)
MEG3_pos5	0.35 (-0.76, 1.45)	1.07 (0.06, 2.08)	PEG3_pos10	0.11 (-0.71, 0.94)	0.19 (-0.43, 0.81)
MEG3_pos6	0.94 (-0.53, 2.41)	1.07 (0.02, 2.13)	PEG3_mean	0.03 (-0.70, 0.75)	0.16 (-0.32, 0.65)
MEG3_pos7	0.55 (-0.62, 1.73)	0.73 (-0.02, 1.48)	SGCE/PEG10_pos1	1.83 (0.72, 2.94)	-0.50 (-1.07, 0.08)
MEG3_pos8	0.66 (-0.71, 2.03)	0.65 (-0.26, 1.55)	SGCE/PEG10_pos2	1.52 (0.34, 2.69)	-0.49 (-1.07, 0.09)
MEG3_mean	0.82 (-0.45, 2.09)	0.97 (0.00, 1.94)	SGCE/PEG10_pos3	1.79 (0.47, 3.10)	-0.39 (-0.91, 0.13)
MEG3-IG_pos1	0.30 (-0.54, 1.14)	0.55 (-0.04, 1.14)	SGCE/PEG10_pos4	1.61 (0.53, 2.70)	-0.78 (-1.48, -0.08)
MEG3-IG_pos2	0.51 (-0.31, 1.34)	0.10 (-0.45, 0.65)	SGCE/PEG10_pos5	1.71 (0.53, 2.88)	-0.90 (-1.70, -0.10)
MEG3-IG_pos3	1.20 (0.14, 2.26)	0.48 (-0.27, 1.23)	SGCE/PEG10_pos6	1.45 (0.09, 2.81)	-0.35 (-1.03, 0.33)
MEG3-IG_pos4	0.94 (0.00, 1.88)	0.12 (-0.60, 0.83)	SGCE/PEG10_mean	1.66 (0.52, 2.80)	-0.56 (-1.13, -0.00)
MEG3-IG_mean	0.74 (-0.08, 1.56)	0.31 (-0.16, 0.79)	PLAGL1_pos1	0.65 (-0.69, 1.98)	-0.98 (-2.17, 0.22)
MEST_pos1	0.09 (-1.03, 1.20)	-0.24 (-1.21, 0.73)	PLAGL1_pos2	1.14 (-0.25, 2.52)	-1.11 (-2.37, 0.14)
MEST_pos2	-0.09 (-1.21, 1.03)	-0.17 (-1.10, 0.77)	PLAGL1_pos3	0.24 (-1.30, 1.79)	-1.08 (-2.24, 0.08)
MEST_pos3	0.59 (-0.79, 1.98)	-0.49 (-1.45, 0.46)	PLAGL1_pos4	1.09 (-0.39, 2.58)	-1.31 (-2.47, -0.16)
MEST_pos4	-0.06 (-1.45, 1.34)	0.80 (-0.36, 1.97)	PLAGL1_pos5	0.99 (-0.35, 2.33)	-1.10 (-2.36, 0.17)
MEST_mean	0.13 (-0.99, 1.25)	-0.02 (-0.90, 0.85)	PLAGL1_pos6	0.73 (-0.47, 1.93)	-1.04 (-2.21, 0.14)
			PLAGL1_mean	0.81 (-0.49, 2.11)	-1.10 (-2.25, 0.04)

Supplemental Table 1. Associations of maternal MDA on methylation of CpGs in Imprinted Control Regions. Females and males were separately evaluated with linear regression for associations of maternal MDA on the methylation status of 48 CpGs in the control regions of 9 imprinted gene loci. Effect estimates for CpG methylation were adjusted for breastfeeding at least 3 months, age of child at behavioral assessment, maternal fiber intake, total calories, folate, education, diabetes, obesity, smoking, and age, as well as paternal age and child parity, premature birth, weight, and race.

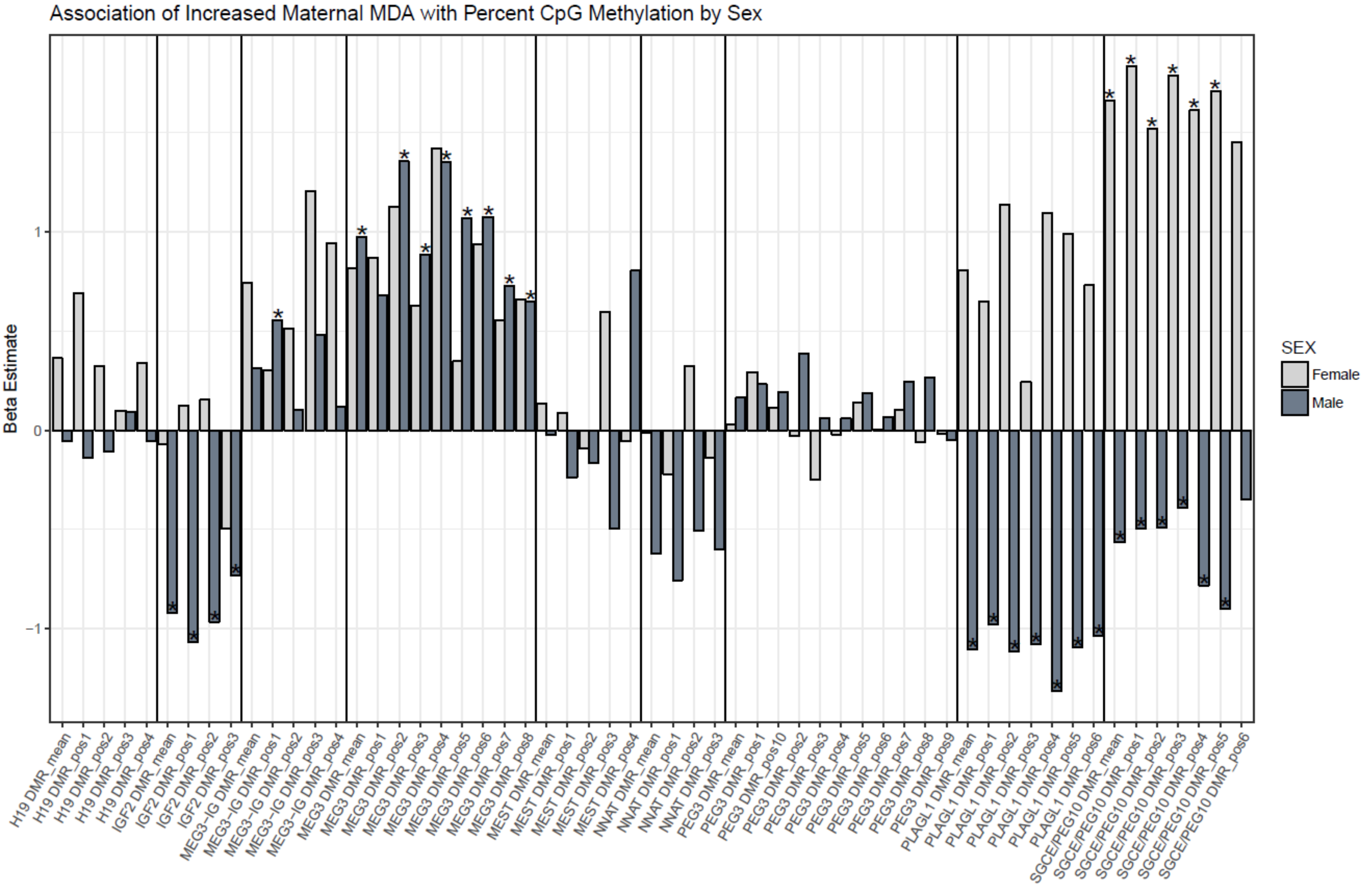

Supplemental Figure 4. Maternal MDA and CpG Methylation. Females (light grey) and males (dark grey) were separately evaluated with linear regression for associations of maternal MDA on the methylation status of 48 CpGs in the control regions of 9 imprinted gene loci. Effect estimates for CpG methylation were adjusted for breastfeeding at least 3 months, age of child at behavioral assessment, maternal fiber intake, total calories, folate, education, diabetes, obesity, smoking, and age, as well as paternal age and child parity, premature birth, weight and race (* FDR < 0.15).

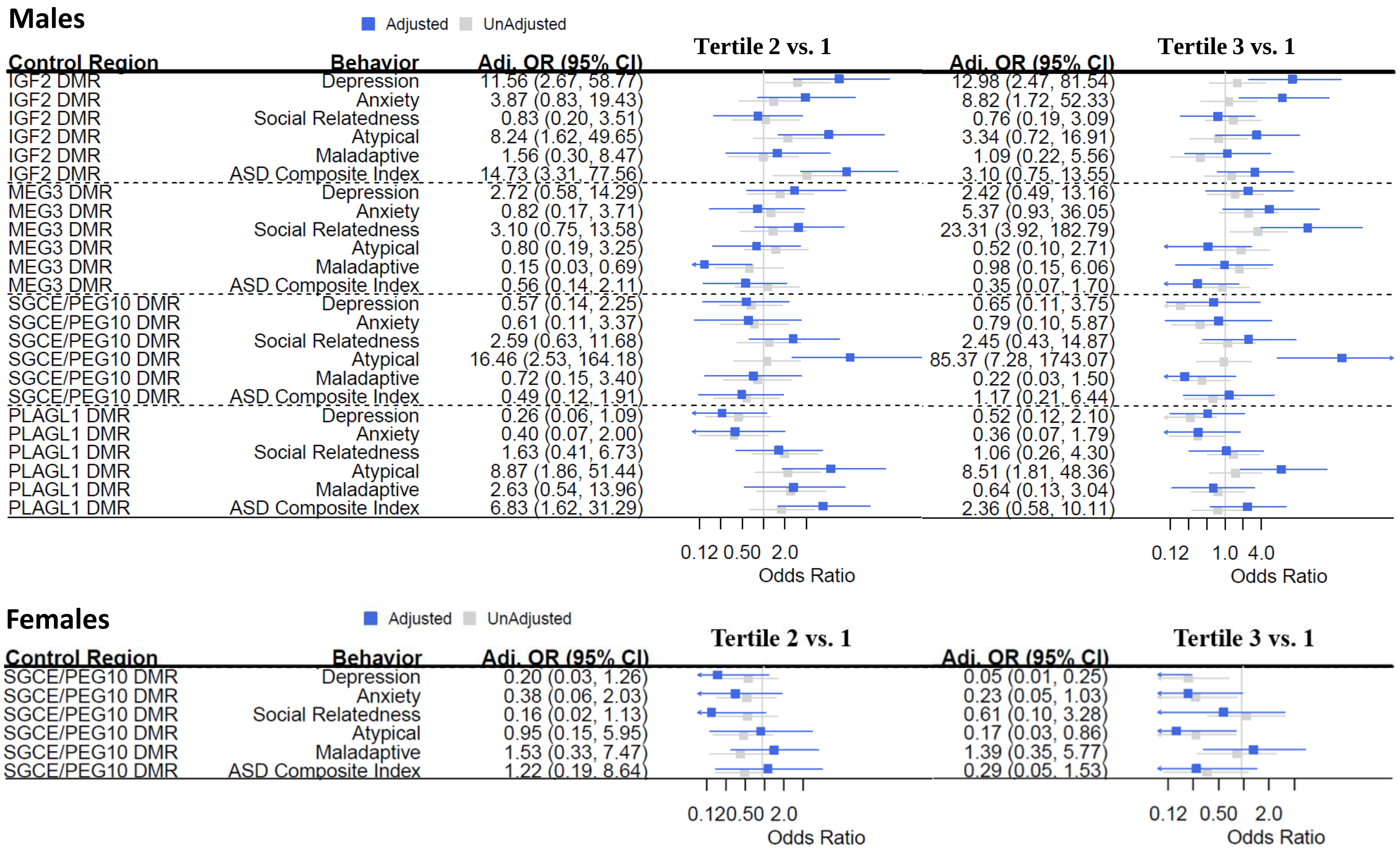

Supplemental Figure 5. Associations of DMR Methylation on Child Behavior by Sex. Tertiles of mean methylation of *IGF2, MEG3, SGCE/PEG10* and *PLAGL1* DMRs were examined for associations with tertiles of child behaviors with adjusted (blue) and unadjusted (grey) ordinal logistic regression. Adjusted odds ratios and 95% CI are reported for the associations of DMR tertile exposure on the likelihood of being in a higher tertile of child behavior. Estimates were adjusted for breastfeeding at least 3 months, age of child at behavioral assessment, maternal fiber intake, total calories, folate, education, diabetes, obesity, smoking, and age, as well as paternal age and child parity, premature birth, weight, and race (n varied between 66 and 82).
